# Ionizing radiation from Chernobyl affects development of wild carrot plants

**DOI:** 10.1038/srep39282

**Published:** 2016-12-16

**Authors:** Zbyszek Boratyński, Javi Miranda Arias, Cristina Garcia, Tapio Mappes, Timothy A. Mousseau, Anders P. Møller, Antonio Jesús Muñoz Pajares, Marcin Piwczyński, Eugene Tukalenko

**Affiliations:** 1 CIBIO/InBIO, Research Center in Biodiversity and Genetic Resources, Associated Laboratory of the University of Porto, Vairão, PT-4485–661 Vairão, Portugal; 2 Department of Biological and Environmental Science, P.O. Box 35, FI-40014 University of Jyväskylä, Finland; 3 Department of Biological Sciences, University of South Carolina, Columbia, SC 29208, USA; 4 Department of Environmental Biology, Chubu University, Kasugai, Aichi 487-8501, Japan; 5 Laboratoire d’Ecologie, Systématique et Evolution, CNRS UMR 8079, Université Paris-Sud, Bâtiment 362, F-91405 Orsay Cedex, France; 6 Chair of Ecology and Biogeography, Nicolaus Copernicus University, Lwowska 1, PL-87-100 Toruń, Poland; 7 Institute of Biology, Taras Shevchenko National University of Kyiv, UA-03022 Kyiv, Ukraine

## Abstract

Radioactivity released from disasters like Chernobyl and Fukushima is a global hazard and a threat to exposed biota. To minimize the deleterious effects of stressors organisms adopt various strategies. Plants, for example, may delay germination or stay dormant during stressful periods. However, an intense stress may halt germination or heavily affect various developmental stages and select for life history changes. Here, we test for the consequence of exposure to ionizing radiation on plant development. We conducted a common garden experiment in an uncontaminated greenhouse using 660 seeds originating from 33 wild carrots (*Daucus carota*) collected near the Chernobyl nuclear power plant. These maternal plants had been exposed to radiation levels that varied by three orders of magnitude. We found strong negative effects of elevated radiation on the timing and rates of seed germination. In addition, later stages of development and the timing of emergence of consecutive leaves were delayed by exposure to radiation. We hypothesize that low quality of resources stored in seeds, damaged DNA, or both, delayed development and halted germination of seeds from plants exposed to elevated levels of ionizing radiation. We propose that high levels of spatial heterogeneity in background radiation may hamper adaptive life history responses.

Classical life history theory predicts that organisms will allocate resources to different life history traits, characteristics that affect ontogeny, reproduction and survival, to optimize overall fitness^1^. Life history traits are shaped by an interaction between extrinsic ecological factors affecting fitness, and intrinsic genetic constraints^2^. Understanding how the environment affects growth, survival, reproduction, and hence fitness, allows for prediction of the evolution of life history strategies^3^. However, environmental heterogeneity, even on a very local scale, generates heterogeneous fitness optima and affects intra-population variation in life histories^4^. Thus environmental heterogeneity can maintain variation in characters and reveal trade-offs between life history traits^5^.

A life history trade-off occurs when an increase in fitness due to a change in one trait is opposed by a decrease in fitness caused by another, functionally linked trait^6^. While trade-offs in life history characteristics play crucial roles in shaping evolutionary trajectories, it is often unclear why trade-offs persist. A possible mechanism maintaining polymorphism in life histories is spatial heterogeneity that provides an opportunity for maintaining different optima in fitness-related traits with decreasing predictability of environmental variation^5^. Environmental heterogeneity can promote different combinations of life history traits and generate trade-offs even at spatially local scales^4^. Consequently, a single optimal life history strategy might be challenged^7^. A recent study suggests that even among close relatives the trade-off between growth and maintenance can be mediated by an increased level of oxidative stress^8^, the imbalance between production of oxygen species during normal physiological processes and the level of antioxidant defence^8^.

In plants trade-offs are often related to resource limitation that constrains expenditure on defence against herbivores and other stressors, or that can be allocated to growth and development^9^. This has potential consequences for adaptive and plastic responses. For example, contamination, such as found in Chernobyl and Fukushima^10^,^11^, can affect both growth rate and survival^12^, as exposure to even low, but chronic ionizing radiation can cause increased oxidative damage and decreases in antioxidant defences^13^. In the Chernobyl area, which is contaminated mainly by caesium-137, developmental instability was found in three plant species that showed a gradual increase in fluctuating asymmetry and frequency of phenodeviants with increasing radiation^14^. Moreover, oxidative stress and ionizing radiation has been linked to a variety of detrimental effects^13^, including elevated mutation rates^15^ and aberrations in the development of pollen^16^, and has been shown to affect gene expression and epigenetic regulation of responses to stress during plant ontogeny^17^. Experimental investigations of rice in the Fukushima region revealed strong transcriptomic effects through activation of genes involved in DNA repair and of defence/stress responses following exposure of seedlings to radiation in contaminated fields^18^,^19^. The activation of DNA repair and anti-stress mechanisms suggested that resources in seedlings were reallocated from development to defence pathways, although the authors did not provide any measures of developmental costs^18^. The hypothesis that plant development can indeed be constrained in contaminated areas due to radiation exposure^20^ comes from research showing that Scots pine *Pinus sylvestris* growth has decreased after the nuclear disaster in Chernobyl^21^. Intriguingly, the response in tree growth was also influenced by other environmental and phenotypic traits suggesting a complex interaction between life-history traits related to developmental and environmental stress caused by ionizing radiation^21^. Nonetheless, experimental tests using common garden approaches are required to verify that exposure to chronic ionizing radiation are indeed responsible for the effects observed in wild populations.

The Chernobyl nuclear accident (26 April, 1986) contaminated vast areas of Europe, but parts of Ukraine, Belarus and Russia were particularly hard hit^11^,^22^. Even within the Chernobyl Exclusion Zone, the limited-entry area around the power station, deposition of radionuclides was highly heterogeneous, resulting in levels of ionizing radiation that varied by five orders of magnitude within relatively short distances of a few hundred meters. Such heterogeneity in radiation levels can cause intra-population variability in selection, potentially preventing the evolution of adaptations to mitigate the elevated oxidative stress caused by ionizing radiation^23^ depending on the amounts of gene flow among metapopulaitons. Here, we conducted controlled common garden experiment under benign green house conditions for wild plants that originated from sites that differed in exposure to radioactive contamination by a factor of almost 400 (Table S2). We tested for the importance of exposure to radioactive contamination of maternal plants on development of the following generation, from germination of seeds until the development of several leaves in young plants. We investigated growth rate between several distinct developmental stages to test if the level of exposure to radioactive contamination, and stored resources accumulated in seeds, affected life history characteristics and potential trade-offs between them. In plants, the seedling stage is often a demographic bottleneck in a species’ life history being particularly vulnerable to resource limitations and herbivore attacks^24^. Embryos of wild carrots *Daucus carota*, subsp. *carota*, the model species studied here, develop slowly over several weeks^25^, thus being vulnerable to environmental stress early during the developmental period^26^. Carrot seedlings usually develop in autumn, and they are subsequently vernalized during winter to induce flowering the next summer^27^. Consequently, we hypothesized that seed germination would be particularly susceptible to the negative effects of radiation and that subsequent developmental stages would be affected by the previous ones. Additionally, we expected that measurable impacts of ionizing radiation would decrease at later developmental stages, partly because sensitive individuals would be purged from the population during the previous developmental periods (i.e. purifying selection), with older plants being less sensitive to radiation as a consequence of this selection.

## Results

### Radioactive exposure levels

Radiation levels measured with a hand-held dosimeter were strongly positively correlated with measures of exposure from soil samples with gamma dosimetry (r = 0.93, p < 0.0001). Also, gamma radiation measurements indicated marked accumulation of radionuclides in carrot roots and above ground parts (Table S1), and significantly correlated with results from a hand-held dosimeter (root: r = 0.63, p = 0.002; above ground plant parts: r = 0.56, p = 0.009; Figure S1). Gamma radiation measurements of soil, roots and plants were also strongly positively correlated (soil-root: r = 0.68, soil-plant: r = 0.63 and root-plant: r = 0.83, p < 0.002). Radiation levels at locations of seed sampling, as measured with a hand-held dosimeter, varied from 0.08 to 30.2 μGy/h with mean and median of 4.83 and 0.50 μGy/h, respectively (Table S2). Estimated exposure to ground contamination varied from 30.9 to 11648.6 kBq/m^2^ (with mean and median of 1862.9 and 192.9 kBq/m^2^) for Cs137, the main radionuclide in the area. Seed mass varied between 0.38 and 2.22 mg with mean and median of 1.04 and 1.06 mg, respectively. Seed mass was not affected by radiation level received by the maternal plant (Pearson partial correlation controlled for the identity of maternal plants, r = −0.04, p = 0.28; Table 1).

### Germination success and time

**Figure 1:**
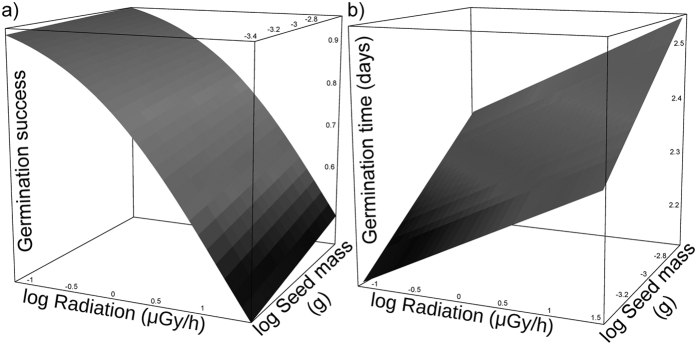
Effects of radioactive contamination (Radiation) and seed mass on early stages of development: (**a**) germination probability and (**b**) germination time in carrots from the Chernobyl Exclusion Zone grown in an uncontaminated greenhouse, in common garden experiment.

Of the 660 seeds collected from 33 maternal plants (20 seeds per plant), 503 (76.2%) germinated successfully. One plant germinated no seeds at all and five germinated the complete set of 20 seeds planted (Table S2). The probability of germination was only affected by the level of radioactive contamination at the location that the maternal plants were collected (z = −949.7, p = 0.0019), while it was not significantly affected by seed mass or its interaction with radiation (interaction was removed from final model, separate model indicated: z = 0.620, p = 0.8; Table 1, Fig. 1a; see also supplementary information: Table S3). Germination success was on average 39% higher (interquartile range) for seeds from the 1^st^ than from the 3^rd^ quartile of the radioactivity distribution (Pearson partial correlation between radiation and germination, controlling for common mother of seeds: r = −0.20, p < 0.0001). Both the level of radioactive contamination (z = 3.37, p = 0.0011) and seed mass (z = 2.22, p = 0.026) affected number of days required for germination (Table 1, Fig. 1b). Seeds originating from plants exposed to higher levels of radioactive contamination (r = 0.21, p < 0.0001), and heavier seeds (r = 0.10, p = 0.022), germinated later than seeds originating from less contaminated areas, and from lighter seeds. The interaction between seed mass and radiation was non-significant.

### Development of cotyledons

Of the 503 seedlings, 490 developed cotyledons (97.4%). The probability of cotyledon emergence was significantly related to germination time (z = 58.73, p < 0.0001; Table 1). Plants that germinated earlier (r = 0.85, p < 0.00001) also developed cotyledons faster. The level of radiation correlated with the time to cotyledon emergence (r = 0.19, p = 0.00002), but the effect of radiation did not significantly influence the time to cotyledon emergence or seed mass in a mixed model (z = 1.68, p = 0.09; Table S3).

### Development of leaves

**Figure 2:**
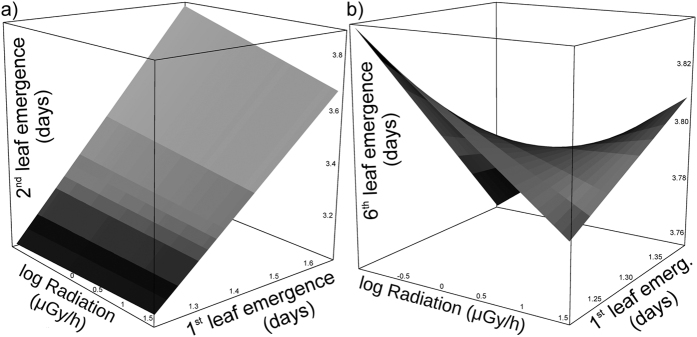
Effects of interactions between radioactive contamination (Radiation) and earlier stage of development (time of development of the 1^st^ leaf) on the time of development of 2^nd^ (**a**) and 6^th^ (**b**) leaves in carrots from the Chernobyl Exclusion Zone grown in an uncontaminated greenhouse, in common garden experiment.

**Figure 3:**
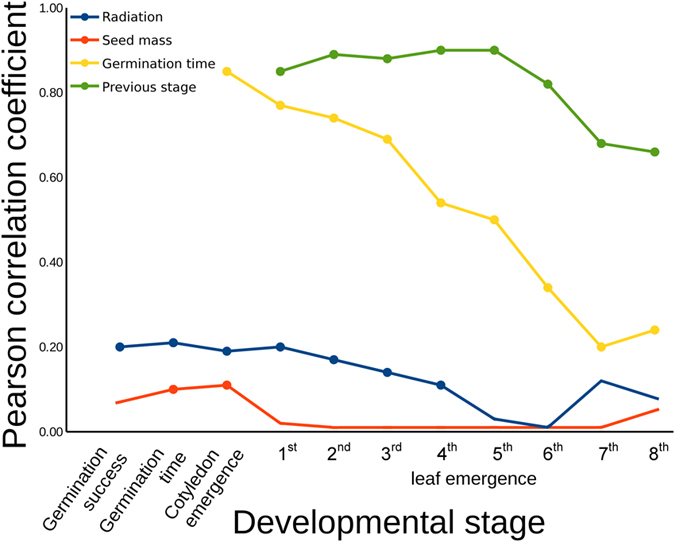
Partial Pearson correlations (accounting for common mother affiliation of seeds) between developmental stages and radioactive contamination (Radiation), seed mass, early (Germination time) and previous stages (Previous stage) on subsequent developmental stages. Dots indicate significant correlations. Correlations between Radiation and Germination are presented as absolute values (it was negative and indicated seeds from highly radioactive locations have low probability of germination).

At the date the experiment was terminated (10 December 2015), the following number of plants developed the following number of leaves: 1^st^ leaf was developed by 482 plants, 2^nd^ by 481, 3^rd^ by 472, 4^th^ by 460, 5^th^ by 444, 6^th^ by 372, 7^th^ by 199, 8^th^ by 81, 9^th^ by 39, 10^th^ by 26, 11^th^ by 12, 12^th^ by 4 and 13^th^ by 4. Seed mass did not significantly affect the emergence of leaves (p > 0.05), except for the 1^st^ leaf, where it interacted with the germination time and cotyledon emergence time (Table 1 and S3). When including the level of development of previous stages in the models, the level of radioactive contamination always showed a significant effect (except for 1^st^ and 3^rd^ leaves), either as a main effect (for 5^th^ leaf) or in interaction with previous developmental stages (for leaves 2, 4, 6 and 7; Table 1, Table S3, Fig. 2). Even when considering the non-independence of multiple tests for 7 developed adult leaves, Bonferroni-corrected tests were significant for radiation level for leaves 2, 4, 5 and 6. The significant correlation between radiation level and the emergence time of adult leaves progressively became weaker with developmental stage (1^st^ leaf: r = 0.21, p < 0.00001, 2^nd^ leaf: r = 0.17, p = 0.0002, 3^rd^ leaf: r = 0.13, p = 0.004, 4^th^ leaf: r = 0.11, p = 0.017) until reaching low and non-significant levels (5^th^ leaf: r = 0.03, p = 0.51, 6^th^ leaf: r = 0.01, p = 0.84, 7^th^ leaf: r = −0.08, p = 0.49; Fig. 3). However, the direct effects of radiation were masked by higher-order interactions in most mixed models (Table 1, Fig. 2).

### Differences between developmental stages

Seeds from mothers exposed to, on average, lower levels of radiation germinated better (means for undeveloped and developed: x_undeveloped_ ± SE = 7.65 ± 0.79, x_developed_ = 3.95 ± 0.38; t = −8.50, p < 0.0001), and developed better between 2^nd^ and 3^rd^ (x_undeveloped_ = 12.84 ± 4.75, x_developed_ = 3.83 ± 0.38; t = −2.72, p = 0.007) and between 3^rd^ and 4^th^ leaf developmental stages (x_undeveloped_ = 9.33 ± 3.82, x_developed_ = 3.68 ± 0.38; t = −1.97, p = 0.049). Significant differences between cumulative averages of plants that did not develop and those that developed persisted until the 7^th^ leaf (t ≤ −2.85, p ≤ 0.0046). Also smaller seeds germinated better (x_undeveloped_ = 0.00112 ± 0.00003, x_developed_ = 0.00102 ± 0.00001; t = −2.87, p = 0.004), and tended to have higher probability of developing cotyledons (x_undeveloped_ = 0.00122 ± 0.00012, x_developed_ = 0.00102 ± 0.00001; t = −1.94, p = 0.052).

## Discussion

We found that chronic exposure to ionizing radiation impacted early-stage development of wild plants by reducing the ability of seeds to germinate or delaying their development. Thus, maternal radiation exposure had statistically significant negative impacts on the probability of seed germination (Fig. 1a). Moreover, time of germination was also delayed as a consequence of elevated radiation exposures (Fig. 1b). We did not find a correlation between seed mass and radioactivity suggesting that pericarp (fruit wall) and endosperm developed normally in plants from contaminated locations, as these structures constitute almost the entire mass of the fruit. However, a relatively long embryonic development in carrots^25^ is a critical step in which exposure to stressful conditions is usually the most damaging and it may negatively impact complete plant development. Indeed, the results of our common garden experiment indicated that early stages of development of embryos (and their germination) are directly and negatively affected by the level of radioactive contamination at the site where maternal plants grew and developed seeds (Fig. 1; Table 1). As expected, such early development also had strong negative effects on later stages of ontogeny of adult leaves (Fig. 2), with likely consequences for overall performance of adult plants^14^,^21^. Moreover, strong negative effects of radioactive contamination early during development may result in selective mortality before later stages and adulthood^28^,^29^. Detecting strong effects of radiation on early stages of ontogeny may have important outcomes for studies aiming to test the consequence of ionizing radiation focused only on adult individuals^21^,^30^. These findings are particularly important as they suggest that previous studies conducted mostly on adults^31^ have likely underestimated radiation effects compared to those that include consequences for earlier stages of development^32^ and across generations.

Our results also suggest that radiation can impact life history trade-offs related to growth rate and ontogeny of distinct life stages^33^. We showed significant variability among individuals in vulnerability to radioactive contamination as some plants are able to develop faster and experience earlier emergence of particular ontogenetic stages, at least during early life, while others are not. However, individuals that develop slower and have later emergence of particular ontogenetic stages can either have slower or faster development of the next leaf, as a response to radioactivity by previous developmental stage interactions (Fig. 2a). Such an effect suggests the fast development might cause trade-offs in ontogeny under stressful conditions^34^, resulting in a complex distribution of leaf emergence times observed in our experiment (Fig. 2b). Therefore, we hypothesize that development-radiation interactions can hamper fitness optimization in the Chernobyl area. This is because ionizing radiation can cause spatially variable levels of oxidative stress, where protective mechanisms would compete for resources with other life history traits to a variable extent, depending on the level of local exposure^12^. Therefore, radiation in general decreases fitness, but the timing of ontogenetic stages are modified by the environment^5^ and are highly dependent on the timing of early ontogeny.

The observed response to radiation can be both genetically adaptive^35^,^36^ and phenotypically plastic^37^. The common garden design such as that employed here can be used to detect genetically based differences in phenotypes among populations. In our case, because early seed development occurs on the maternal plants, which were exposed to varying environmental conditions, we are unable to test for genetic differences. Such tests would require multi-generational experiments under common garden conditions^38^ overcoming across generation maternal effects, persistent even after environmental specific effects are removed^39^. Therefore, our findings relate to the direct effects of radiation on developing seeds as expressed by germination responses, and the effects of maternal genotype, phenotype and environment (e.g. radiation exposure) on developing seeds and their interaction effects on developing seedlings.

In conclusion, in our experiment we found that maternal exposure to ionizing radiation had persistent negative effects on the ontogeny of progeny. The effect seems to be strongest during early development (i.e. germination), and persists as a complex developmental trajectory that depends on the timing of early ontogenetic stages. We hypothesize that low quality of resources stored in seeds, damaged DNA, epigenetic factors or their combination may delay development, and in some cases completely halt germination in seeds from plants exposed to elevated ionizing radiation. Multigenerational common garden and transplant experiments will be necessary to determine if the first generation variation observed under common garden conditions has a genetic basis (i.e. reflects the effects of natural selection) and has any adaptive significance. Moreover, as wild carrots accumulate substantial amounts of radionuclides in tissues, this shows great promise as a useful model system for investigating the genetic, ecological and evolutionary consequences of long-term radioactive contamination in the Chernobyl region.

## Material and Methods

### Study design

Wild carrots (*Daucus carota* subsp*. carota,* Apiaceae) were used for this study. This species is a common weed throughout temperate regions of the world. For the experiment, seeds were collected from a population growing at abandoned field (51°21′N, 30°0′E, September 2012) located 7.5 km SW of the destroyed reactor 4 of the Chernobyl nuclear power plant^11^,^40^, an area characterized by very local heterogeneous levels of radioactive contamination. This local scale of variation of exposure to radioactive contamination is not visible on the general overview maps (e.g. http://radatlas.isgeo.com.ua/). The only way to depict such local variation is conducting localized hand dosimetry measurements. Radiation measurements of the soil were made exactly under focal plants to estimate exposure of particular plants to radioactive particles using hand-held dosimeters placed on the ground (Inspector, International Medcom, Sebastopol, CA, USA) and calibrated to measure Gray (Gy). The ground level measurement of α, β, and γ radiation with a hand-held dosimeter provide highly reliable field measurements of level of radiation^11^, and in plants it can be related to both external and internal potential dose exposures (Figure S1). The radioactive exposure per unit time (μGy/h), rather than cumulative dose exposure, was used as predictor in all analyses to avoid artificial inflation of exposure differences between parental plants caused by spurious estimation of unknown vegetative period of a particular plant. Prior to the common garden (same environment) experiment in the greenhouse, collected seeds were weighed in groups of 5, and this total mass was divided by five to obtain individual mean seed mass. We randomly selected 660 seeds originating from 33 maternal plants (20 seeds per plant) collected across a radiation gradient, and these seeds were subsequently planted. Seeds were placed on the soil surface in individual pots after an initial 3-day cold treatment at 5 °C, simulating natural conditions prior to germination. Pots were randomly distributed and randomized daily by mixing the position of at least 30 pots to homogenize germination/growing conditions. The greenhouse was equipped with an automatic water vaporiser, and thus humidity (but not temperature) was controlled throughout the experiment, starting from 5 October 2015. The greenhouse was inspected daily (excluding Sundays) until 10 December 2015 and dates of emergence of subsequent developmental stages were recorded: emergence of radicle (indicator and timing of successful germination), cotyledons and subsequent adult leaves, respectively.

### Dosimetry

Samples from 21 plants were used to estimate accumulation of radionuclides in soil, root and plants above ground parts, using gamma spectrometry. Activity of 137Cs, in 63 soil samples (3 per location of sampled carrots), 21 above ground parts of carrots and 21 carrot roots, was measured with the Berkeley Nucleonics SAM 940 radionuclide identifier system equipped with a 10 × 10 × 10 cm NaI detector. This detector was chosen because of its very high efficiency allowing for very rapid measurements of gamma emitters. The detector was enclosed in 5–10 cm thick lead shielding (about 400 kg) to reduce the noise from background radioactivity. Given the high sensitivity and large amounts of shielding, we are able to accurately measure radioactivity levels to less than 1 or 2 bq per sample (i.e. calculated errors are typically around 0.5 bq). The system was calibrated with reference standard sources. Samples of soil, and entire roots and plants were measured separately for 120 s (soil) and 180 s (roots and plants, longer measures up to 900 s gave similar results). With corrections for laboratory background, the activity of 137Cs was evaluated. Samples were weighed prior to measurements on a Radan electronic balance and individual mass was used to standardize radioactivity across samples. Estimations of exposure to ground contamination for Cs137 were recalculated from soil dose-rates^41^.

### Statistical analyses

Generalized linear mixed model analyses were used to test for effects of contamination level and seed mass on germination success and time, and time of subsequent developmental stages. The identity of the maternal plant was included as a random effect in all models to control for non-independence of related seeds. Separate models were tested for germination rate, time of germination, and time of emergence of cotyledons and subsequent adult leaves. Due to the large number of tested iterations, data sets with a large number of observations (>100, until 7^th^ leaf) were included in the analyses. The first model included a binary dependent variable describing germination of seeds, or lack there-of. Subsequent models included time of development of a particular stage since seeding as the dependent variable. Models for a given developmental stage were built hierarchically to include information about previous stages. Basic initial models included seed mass, radiation level at the collection location, and interaction between these variables, and for models other than germination success and time of the previous developmental stages and the interaction between those and seed mass and radiation level, respectively. For statistical tests a non-linear mixed model procedure was used in the R (version 3.2.3) package “lme4” with the glmer function and Laplace approximation (although Adaptive Gauss-Hermite Quadrature, nAGQ = 50, gave similar results)^42^. A bimodal distribution with logit link function was applied to analyse germination success, and a Gaussian with log link function (lognormal) was used for all developmental time analyses. All independent variables were log transformed prior to analysis to reduce the kurtosis of the distribution of radiation and seed mass. Statistical significance of the effects, and backward model reductions (selection of fixed effects), were performed by log likelihood ratio test with the “LMERConvenienceFunctions” R package. Differences in mean radiation and seed mass of individuals surviving between developmental stages were also tested.

## Additional Information

**How to cite this article**: Boratyński, Z. *et al*. Ionizing radiation from Chernobyl affects development of wild carrot plants. *Sci. Rep.* **6**, 39282; doi: 10.1038/srep39282 (2016).

**Publisher's note:** Springer Nature remains neutral with regard to jurisdictional claims in published maps and institutional affiliations.

## Supplementary Material

Supplementary Results

## Figures and Tables

**Table 1:** Mixed model results of the effects of radioactive contamination (Radiation) and seed mass (and earlier stages of development) on various progressive developmental stages of wild carrots collected as seeds from the Chernobyl Exclusion Zone (see Table S3 for results for emergence time of leaves 1, 3–5 and 7).

Dependent variables	Model Log-Likelihood	Effect	Estimate	SE	value	pLLRT#
Germination	−273.3	Radiation	−1.2321	0.001	−949.7	0.0019
Germination time	−436.1	Radiation	0.0773	0.023	3.37	0.0011
		Seed mass	0.2867	0.129	2.22	0.026
2^nd^ leaf emergence	−917.3	1^st^ leaf	0.2173	0.418	0.52	NA
		Cotyledon	1.5753	0.360	4.37	NA
		Radiation	0.2711	0.052	5.17	NA
		Seed mass	0.3576	0.270	1.32	NA
		1^st^ l. * Rad.	−0.2180	0.040	−5.47	<0.00001***
		1^st^ l. * Mass	−1.3547	0.398	−3.40	0.0202
		Cot. * Mass	1.2002	0.349	3.43	0.0139
6^th^ leaf emergence	−802.0	1^st^ leaf	−0.1867	0.066	−2.82	NA
		4^th^ leaf	0.3833	0.131	2.92	0.009
		5^th^ leaf	1.7129	0.119	14.35	<0.00001***
		Radiation	−0.3048	0.087	−3.50	NA
		1^st^ l. * Rad.	0.2355	0.069	3.44	0.0007**

Values - “z” for Germination model assuming logit link function, “t” for the rest models assuming log link function. NA - part of higher-order interaction, LLRT skipping term. Terms significant after applying Bonferroni corrections for multiple separate tests for 7 leaves: ^***^<0.00001, ^**^0.00001–0.01. ^#^Interactions with pLLRT > 0.04 were progressively excluded from presented model.
