# Supplementary material for: Ionizing radiation from Chernobyl affects development of wild carrot plants
Source: Sci Rep. 2016 Dec 16;6:39282. doi: 10.1038/srep39282 (PMC5159907; doi:10.1038/srep39282)
Supplement: Supplementary Results [file srep39282-s1.pdf]

Ionizing radiation from Chernobyl affects development of wild carrot plants

Zbyszek Boratyński[1,2], Javi Miranda Arias[1], Tapio Mappes[2], Timothy A. Mousseau[3,7], Anders P. Møller[4], Antonio Jesús Muñoz Pajares[1], Cristina Garcia[1], Marcin Piwczyński[5], Eugene Tukalenko[2,6]

[1] CIBIO/InBIO, Research Center in Biodiversity and Genetic Resources, Associated Laboratory of the University of Porto, Vairão, PT-4485–661 Vairão, Portugal

[2] Department of Biological and Environmental Science, P.O. Box 35, FI-40014 University of Jyväskylä, Finland

[3] Department of Biological Sciences, University of South Carolina, Columbia, SC 29208, USA

[4] Laboratoire d'Ecologie, Systématique et Evolution, CNRS UMR 8079, Université Paris-Sud, Bâtiment 362, F-91405 Orsay Cedex, France

[5] Chair of Ecology and Biogeography, Nicolaus Copernicus University, Lwowska 1, PL-87-100 Toruń, Poland

[6]Institute of Biology, Taras Shevchenko National University of Kyiv, UA-03022 Kyiv, Ukraine

[7] Department of Environmental Biology, Chubu University, Kasugai, Aichi 487-8501, Japan

Table S1. Results from hand dosimeter substrate measures and gamma dosimetry soil, plant and root measurements.

	Mean	SD
Substrate radiation µSv/h	5.64	7.55
Soil radionuclide exposure Bq/kg	23296.24	28691.02
Plant radionuclide accumulation Bq/kg	730.08	971.68
Root radionuclide accumulation Bq/kg	1480.67	2068.41

Table S2. Ionizing radiation in the location of maternal plant collection and the number of germinated seeds per plant.

Maternal plant	Radiation (µGy/h)	Germinated seeds
1	6.10	1
2	3.60	2
3	3.44	17
4	5.39	14
5	4.42	0
6	0.50	11
7	0.50	15
8	0.50	18
9	0.51	20
10	0.40	15
11	0.30	19
12	0.30	14
13	0.30	19
16	21.40	18
17	21.63	16
18	25.78	6
19	28.30	12
20	30.20	15
21	0.17	19
22	0.16	17
23	0.19	17
24	0.17	20
25	0.17	19
26	0.67	18
27	1.02	20
28	1.10	18
29	1.05	19
30	0.50	16
31	0.14	20
32	0.10	20
33	0.08	18
34	0.15	13
35	0.14	17

Table S3. Mixed model results (based on Laplace approximation) of the effects of ionizing radioactive contamination and seed mass (and earlier stages of development) on various subsequent developmental stages of wild carrots collected as seeds from the Chernobyl Exclusion Zone and grown in a greenhouse. Reduced results are presented in Table 1. The significance levels of effects were estimated with log likelihood ratio test (pLLRT).

Dependent variables	Model Log-Likelihood	Effect	Estimate	SE	value	pLLRT
Germination	-273.3	Radiation	-1.2321	0.001	-949.7	0.0019
Germination time	-1436.1	Radiation	0.0773	0.023	3.37	0.0011
		Seed mass	0.2867	0.129	2.22	0.026
Cotyledon emergence	-1111.3	Germ. time	1.9236	0.0257	74.85	<.00001
		Radiation	0.0124	0.007	1.68	0.094
		Seed mass	0.0777	0.043	1.81	0.080
1st leaf emergence	-969.6	Germ. time	-1.2934	0.422	-3.06	NA
		Cotyledon	2.5703	0.383	6.72	NA
		Seed mass	0.2842	0.284	1.00	NA
		Germ.* Mass	-1.5877	0.419	-3.79	0.0003**
		Cot. * Mass	1.0665	0.385	2.77	0.0091
2nd leaf emergence	-917.3	1st leaf	-0.0056	0.416	-0.01	NA
		Cotyledon	1.8946	0.372	5.10	NA
		Germination	0.1023	0.050	2.04	NA
		Radiation	0.2066	0.057	3.59	NA
		Seed mass	0.1730	0.277	0.63	NA
		1st l. * Rad.	-0.3069	0.061	-5.00	<.00001***
		1st l. * Mass	-1.5246	0.398	-3.84	0.0109
		Cot. * Mass	1.5658	0.360	4.35	0.0024*
		Germ.* Rad.	1.1183	0.058	2.03	0.0437
		Mass * Rad.	-0.0591	0.025	-2.36	0.0215
3rd leaf emergence	-926.3	Germ. time	0.1082	0.043	2.50	0.0132
		1st leaf	0.4490	0.083	5.43	<.00001***
		2nd leaf	1.3824	0.084	16.43	<.00001***
4th leaf emergence	-881.2	1st leaf	-0.0214	0.068	-0.31	NA
		2nd leaf	0.2325	0.085	2.73	NA
		3rd leaf	1.9047	0.090	21.25	<.00001***
		Radiation	-0.0813	0.075	-1.08	NA
		1st l. * Rad.	-0.2135	0.082	-2.59	0.011
		2nd l. * Rad.	0.2486	0.087	2.84	0.005*
5th leaf emergence	-890.9	3rd leaf	0.6107	0.099	6.17	<.00001***
		4th leaf	1.6724	0.098	17.02	<.00001***
		Radiation	-0.0077	0.003	-2.67	0.0076*
6th leaf emergence	-802.0	1st leaf	-0.1867	0.066	-2.82	NA

		4th leaf	0.3833	0.131	2.92	0.0090
		5th leaf	1.7129	0.119	14.35	<.00001***
		Radiation	-0.3048	0.087	-3.50	NA
		1st l. * Rad.	0.2355	0.069	3.44	0.0007**
7th leaf emergence	-433.1	3rd leaf	-0.1063	0.119	-0.89	NA
		6th leaf	3.0479	0.249	12.24	NA
		Radiation	-0.4946	0.179	-2.76	NA
		Seed mass	-2.5465	0.386	-6.60	NA
		3rd l. * Rad.	0.3349	0.123	2.72	0.0098
		6th l. * Mass	1.5454	0.236	6.55	0.0266

Values - “z” for Germination model assuming logit link function, “t” for the rest of the models assuming log link function. NA - part of higher-order interaction, LLRT skipping term. Terms significant after applying Bonferroni correction for multiple separate tests for 7 leaves: *** <.00001, **.00001-0.01, * 0.01-0.05.

Supplementary Figure:

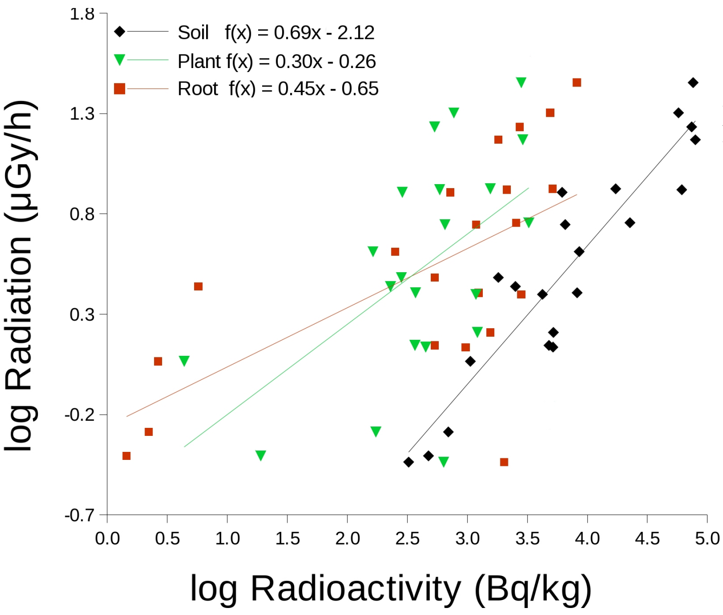

Figure S1. Correlations between hand-held dosimeter measures (log transformed, Radiation) and soil, plant (above ground parts) and root radioactive dose exposure and uptake in carrots (log transformed, Radioactivity).
